# Perianal Abscess and Its Recurrence

**Published:** 1984-04

**Authors:** N. I. Ramus, J. Bradley

**Affiliations:** Department of Surgery, Southmead Hospital, Bristol; Department of Surgery, Southmead Hospital, Bristol


### Perianal Abscess and its Recurrence

**N. I. Ramus, MD FRCS**
**J. Bradley, MB ChB**
Department of Surgery, Southmead Hospital, Bristol

Over half of all anorectal absecsses are situated in the perianal region. Following simple incision and drainage, two-thirds of the patients are said to have recurrent problems, usually a fistula-in-ano.[1-3] A more aggressive approach has therefore been recommended for perianal abscess with a 'lay open' procedure entailing a complete internal sphincterotomy.[4] Because this had not been our clinical impression, and as surgical treatment of perianal abscess is often left to the most junior member of the surgical team, an 11-year review of the results of incision and drainage for perianal abscess in a district general hospital has been carried out prior to any fundamental change in operative technique and thereby surgical teaching.

### MATERIALS AND METHODS

All simple perianal abscesses presenting to Southmead Hospital, Bristol between 1970 and 1980 were included. Ischiorectal, deep intermuscular abscesses and those in association with inflammatory bowel disease were excluded.

All patients had incision and drainage of the abscess under a general anaesthetic. At the time of operation an attempt was made to demonstrate an associated fistula-in-ano. The wound was packed, usually with Eusol and paraffin, and the patient discharged to district nurse care as soon as practicable.

### RESULTS

Three hundred and forty-two patients presented with a perianal abscess in the 11 year reviewed. Only one fistula-in-ano was demonstrated at the initial operation. Forty-five of these patients had developed a recurrent abscess (13%), 35 within two years of the primary incision and drainage. Twenty-six patients had one recurrence (2 fistulae demonstrated), 13 had two recurrences (3 fistulae) and six had three or more recurrences (4 fistulae), representing an overall fistula rate of less than 3%. No recurrence followed treatment of the fistula-in-ano.

### DISCUSSION

Within the limitations of the study with an inevitable loss of some patients with recurrent perianal sepsis to other hospitals in Bristol, we were unable to confirm the high incidence of postoperative complications including fistulae that have been previously reported after incision and drainage for perianal abscess. In this review 87% of patients with perianal abscess were cured by their first operation, and no change in management from simple incision and drainage is therefore required at this stage. There is however an increasing chance of an associated fistula in patients with recurrent perianal sepsis, and more radical procedures may well then be indicated.

### ACKNOWLEDGEMENTS

We thank the consultant surgeons at Southmead Hospital, Bristol, for allowing us to review their patients.

### REFERENCES

1. HUGHES, E. S. R., TURRELL, R. (ed) (1969) Diseases of the colon and anorectum. 2nd edition. Philadelphia: W. B. Saunders Co. pp. 955–996.
2. WAGGENER, H. U., EISEMAN, B. (ed) (1969) Surgical Clinics of North America. Vol. 49. Philadelphia: W. B. Saunders Co. pp. 1227–1233.
3. SCOMA, J. A., SALVATI, E. P., RUBIN, R. J. (1974) Incidence of fistulas subsequent to anal abscesses. *Dis. Colon Rectum* **17**, 357–359.
4. HANLEY, P. H., FERGUSON, J. A. (ed) (1978) Surgical Clinics of North America. Vol. 58. Philadelphia: W. B. Saunders Co. pp. 487–503.

*Address for correspondence*: N I R, Department of Surgery, Bristol Royal Infirmary, Bristol BS2 8HW

